# Association of biomarkers and risk scores with subclinical left ventricular dysfunction in patients with type 2 diabetes mellitus

**DOI:** 10.1186/s12933-022-01711-5

**Published:** 2022-12-09

**Authors:** Amera Halabi, Elizabeth Potter, Hilda Yang, Leah Wright, Julian W. Sacre, Jonathan E. Shaw, Thomas H. Marwick

**Affiliations:** 1 (Dept) Imaging Research, Baker Heart and Diabetes Institute, 75 Commercial Road, PO Box 6492, Melbourne, VIC 3004 Australia; 2 School of Public Health and Preventive Medicine, Monash University, 553 St Kilda Road, Melbourne, VIC 3004 Australia; 3 (Dept) Imaging Research, Menzies Institute for Medical Research, 17 Liverpool Street, Hobart, TAS 7000 Australia

**Keywords:** Diabetes, LV dysfunction, Heart failure, Echocardiography, Natriuretic peptides

## Abstract

**Background:**

Subclinical LV dysfunction (LVD) identifies heart failure (HF) risk in type 2 diabetes mellitus (T2DM). We sought the extent to which clinical scores (ARIC-HF, WATCH-DM), natriuretic peptides (NTpBNP) and troponin (hs-TnT) were associated with subclinical LV dysfunction (LVD). These associations could inform the ability of these tests to identify which patients should undergo echocardiography.

**Methods:**

Participants with T2DM were prospectively recruited from three community-based populations. ARIC-HF risk at 4 years and WATCH-DM scores were calculated from clinical data. NTpBNP and hs-TnT were measured using an electro-chemiluminescence assay. All underwent a comprehensive echocardiogram. We calculated the sensitivity and specificity of clinical scores and biomarkers to identify abnormal global longitudinal strain (GLS ≥ −16%)), diastolic function (E/e’ ≥ 14 or e’ < 8 cm/s), left atrial volume index (LAV > 34 ml/m^2^) and LV hypertrophy (LV mass index > 88 g/m^2^ (F) > 102 g/m^2^(M)).

**Results:**

Of 804 participants (median age 69 years [inter-quartile range (IQR) 65–73], 36% female), clinical scores suggested significant HF risk (median ARIC-HF 8% [IQR 4–12]; WATCH-DM 10 points [IQR 8–12]), and the median NTpBNP was 50 pg/mL [IQR 25–101] and hs-TnT 9.6 pg/mL [IQR 6.8–13.6]. Abnormal GLS was present in 126 (17%), elevated E/e’ in 114 (15%), impaired e’ in 629 (78%), increased LAV in 351 (44%) and LV hypertrophy in 113 (14%). After adjustments for age, body-mass index, and renal function, each standard deviation increase in NTpBNP was associated with a GLS increase of 0.32 (*p* < 0.001) and hs-TnT increase by 0.26 (*p* < 0.001). Similar trends were observed with ARIC-HF (standardised β = 0.22, *p* < 0.001) and WATCH-DM (standardised β = 0.22, *p* < 0.001) in univariable analyses. However, none of the risk assessment tools provided satisfactory discrimination for abnormal GLS (AUC 63%), diastolic indices (e’ AUC 54–61%) or LV mass (AUC 59–67%). At a sensitivity of 90%, there was an unacceptably low (< 50%) specificity.

**Conclusion:**

Although risk assessment based on clinical scores or biomarkers would be desirable to stratify HF risk in people with T2DM, they show a weak relationship with subclinical LVD.

**Supplementary Information:**

The online version contains supplementary material available at 10.1186/s12933-022-01711-5.

## Introduction

Subclinical left ventricular dysfunction (LVD) often pre-dates the development of symptomatic heart failure (HF) [1]. It is under-recognised, as assessment of LV function is usually triggered by symptoms or clinical signs of HF [1]. However, subclinical LVD is not a benign condition [2]; its recognition could trigger prevention strategies that may ultimately reduce the development of HF and death. As universal screening with standard echocardiography has historically not been feasible in people with risk factors for HF because of cost and access, an alternative screening process has been sought for early detection of LVD.

Clinical risk scores for HF offer one option to identify patients for testing for LVD. The Atherosclerotic Risk in Communities (ARIC) HF score is sensitive for the 10-year prediction of symptomatic HF in the general community (AUC = 0.79) [3]. The more recently published WATCH-DM score, specifically tailored to people with type 2 diabetes mellitus (T2DM) has similar discrimination for predicting 5-year risk of HF (AUC = 0.77) [4]. The use of biomarkers, such as N-terminal pro-brain natriuretic peptide (NTpBNP) and high-sensitivity troponin (hs-TnT) is an alternative approach to detect subclinical LVD. The STOP-HF study used a strategy of BNP-guided management to institute preventative therapies and screening with echocardiography to further assess subclinical LVD [5]. Based on this and other published papers on the utility of measuring clinical biomarkers to assess risk of future HF [6–9], the recent American Diabetes Association (ADA) guidelines suggest measuring NTpBNP and hs-TnT in all patients without a history of clinical HF to delineate the need for cardiac imaging [10]. This study aimed to examine the utility of risk scores (ARIC-HF and WATCH-DM) and clinical biomarkers (NTpBNP and hs-TnT) in detecting subclinical LVD or structural heart disease (SHD) in community-based populations with T2DM thus identifying those with stage B HF and at risk of progression to symptomatic HF.

## Methods

### Patient population

This analysis involved 804 people with a prior history of T2DM, recruited from two geographical locations (Tasmania and Victoria, Australia) into 3 community-based studies. The PREDICT study recruited people, aged 18–80 years from Victoria, Australia with a prior history of T2DM. The T2DM cohorts of the Tasmanian and Victorian studies of echocardiographic detection of LV dysfunction (TasELF and VicELF) recruited people aged > 65 years with T2DM, but excluded people with prior history of HF, CAD, or moderate/severe valvular heart disease, estimated glomerular filtration rate (eGFR) < 60 ml/min/m^2^, New York Heart Association functional class > 2 or oncologic life expectancy (< 12 months).

### Clinical data

Demographic and clinical data were verified by interview and included documentation of prior comorbid conditions and current medication. Body mass index (BMI) measurements were calculated by dividing weight (kilograms) by height in metres squared (kg/m^2^). The waist-to-hip ratio (WHR) was calculated by dividing the waist circumference (centimetres) to the hip measurement (centimetres). Haemodynamic data included systolic and diastolic blood pressure (SBP and DBP, respectively [mmHg]), which were recorded with a cuff sphygmomanometer.

Blood samples were collected and included measurement of renal function (creatinine [mmol/L] and estimated glomerular filtration rate [eGFR ml/min/m^2^]), haemoglobin A1c (HbA1c) and fasting plasma glucose (mmol/L). NTpBNP and hs-TnT were measured using electro-chemiluminescence assays (Roche Diagnostics, Rotkreuz, Switzerland).

### Echocardiographic assessment

The same comprehensive echocardiogram protocol was performed in all studies (Acuson SC2000, Siemens, Mountain View, CA; Vivid S70, GE Healthcare, Boston, MA). LV mass (LVM) was measured by 2D-guided M-mode in the parasternal long axis window using measurements at end-diastole (0.8 x [1.04 x (LV internal diameter + interventricular septal diameter + posterior wall diameter)^3^ – (LV internal diameter)^3^ + 0.6 g). LVM was indexed to BSA. LV systolic function was assessed by measurement of 2-dimensional ejection fraction and global longitudinal strain (GLS), which was measured in the 3 standard apical views. In the apical-3 chamber view the operator set the timing of closure of the aortic and mitral valves. The endocardial border was manually traced, and calculation of myocardial deformation was triggered by the R wave on the ECG. The strain values of all LV segments were averaged and reported as GLS (%).

Diastolic function was assessed in the apical 4-chamber view by measurement of the transmitral inflow Doppler (to determine E velocity) and mitral annular tissue velocity by Doppler (reported as the average of the septal and lateral e’). LV filling pressure (E/e’) was then calculated. Left atrial volume (LAV) was measured in the LA focused view of the apical-4 and -2 chamber. LAV was indexed to body surface area (BSA).

### Risk scores

Components of the ARIC-HF risk score included age, gender, race (assumed non-African American), smoking history (current or previous), heart rate (beats per minute), SBP, BMI, prior history of CAD, treatment for hypertension and history of T2DM. The ARIC-HF risk score was recorded in percentage risk of HF at 4 years and was restricted to participants aged over 55 years.

The WATCH-DM risk score was calculated based on age, BMI, SBP, DBP, QRS duration, fasting plasma glucose, high-density lipoprotein (HDL), prior myocardial infarction, and coronary artery bypass grafting. The WATCH-DM risk score was recorded as ‘points’ in the dataset.

### Diabetic cardiomyopathy subtypes

Diabetic cardiomyopathy (DCM) phenotypes were explored amongst the cohort based on echocardiographic data and NTpBNP levels. Previously published work has identified increasingly restrictive diagnostic criteria. The *least restrictive* diagnosis is characterized by at least one echocardiographic abnormality (diastolic dysfunction [presence of E/e’ > 14 or e’ < 8 cm/s], LA enlargement or elevated LV mass), an *intermediate restrictive* diagnosis by at least 2 echocardiographic abnormalities, and the most *severely restrictive* criteria defined by at least 2 echocardiographic abnormalities and elevated NTpBNP level (≥ 125 pg/mL) [11].

### Statistical analysis

A Shapiro-Wilk test was conducted on continuous variables to assess normality. As such, due to non-normal distribution, continuous variables are expressed as median [inter-quartile range; IQR]. Categorical variables are reported as counts (%). A *p* value of < 0.05 was considered statistically significant. Missing HDL data (247 [31%]) were handled by multiple imputation using a linear regression model matched for age, gender, BMI, BP, treatment with a statin and prior history of obesity, hyperlipidaemia, MI, smoking and stroke.

Analysis of echocardiographic parameters were explored by two approaches; either continuous variables or dichotomised into 2 groups (normal and abnormal). The latter was defined as GLS ≥ -16%, E/e’ > 14, e’ < 8 cm/s, LAVI > 34 ml/m^2^ and LVMi > 88 g/m^2^ for female gender or 102 g/m^2^ for males. The prevalence of 3 DCM subtypes were reported as percentage counts. Associations between abnormal echocardiographic parameters, NTpBNP and diabetes duration were performed to explore the progression of subclinical LVD and SHD over time.

Prevalence rates of abnormal clinical biomarker levels based on ADA guidelines were reported (cut-off levels defined as NTpBNP > 125 pg/mL or hs-TnT > 12.5 pg/mL [latter based on manufacturer’s recommendation of the 99^th^ percentile for the assay]) [10].

Linear regression analysis was performed to explore the relationship between echocardiographic parameters as continuous variables and the 3 risk assessment tools. ARIC-HF and WATCH-DM risk scores were explored in univariable regression analyses. Multivariable regression was conducted for NTpBNP and hs-TnT to control for relevant confounding clinical factors (i.e., age, obesity, and renal function). The standardised ß was reported to detect the amount of change in an echocardiographic parameter for each 1 standard deviation increase in the risk assessment tool. This then allowed for comparisons to be drawn between the tools.

The discriminative ability of NTpBNP to detect an abnormal echocardiographic parameter was performed by calculating its sensitivity at incremental levels of NTpBNP, starting at 50 pg/mL. To further evaluate the ability of the 4 risk assessment tools to detect an abnormal echocardiographic parameter, a 90% cut-point for sensitivity was set and the corresponding risk score (ARIC-HF or WATCH-DM) and clinical biomarkers (NTpBNP > 125 pg/mL and hsTnT > 12.5 pg/mL) levels were reported. The specificity at the 90% sensitivity was then reported (%). The area under the receiver operator characteristic (ROC) curve was calculated for each risk assessment tool. All statistical analyses were performed using Stata Version 16.1 (StataCorp LLC, College Station, TX).

## Results

### Patient characteristics

A total of 804 participants with T2DM (median age 69 years [IQR 65, 73], 287 (36%) women) were identified (Table 1). Median diabetes duration was 10 years [5, 17]. The most common HF risk factor other than T2DM was overweight and obesity. The ARIC-HF score indicated a median 4-year risk of HF of 8% [IQR 4, 12%], and the WATCH-DM score (11 points [9, 13]) was analogous. Median NTpBNP and hs-TnT were within the normal range (50 pg/mL [25, 101] and 9.4 pg/mL [6.8, 13.6]). Most participants were on ACEi/ARB (542, 67%), statin (552, 69%) or metformin therapy (618, 77%). Demographic data for each sub-population studied are displayed in the Additional file 1: Appendix S1.

**Table 1:** Demographic data for the cohort of participants with T2DM (n = 804)

Variable	Median [IQR] or n (%)
Age (years)	69 [65, 73]
Female gender	287 (35.7)
Diabetes duration (years)	10 [5, 17]
Overweight and obese	694 (86.4)
Prior MI	62 (7.7)
Prior stroke	48 (6)
History of smoking (current & ex)	386 (48)
BMI	29.3 [26.4, 33.2]
WHR	0.98 [0.92, 1.03]
SBP (mmHg)	131 [120, 142]
DBP (mmHg)	74 [67, 82]
HbA1c (%)	7.2 [6.5, 8.5]
Creatinine (mmol/L)	76 [65, 92]
eGFR (ml/min/1.73m^2^)	84.0 [70, 93]
HDL (mg/dL)	46 [39, 56]
NTpBNP (pg/mL)	50 [25, 101]
hs-TnT (pg/mL)	9.4 [6.8, 13.6]
Medications	
Beta-blocker	119 (14.8)
ACEi/ARB	542 (67.4)
Diuretics	159 (19.8)
CCB	222 (27.6)
Statin	552 (68.7)
Anti-platelet	295 (36.7)
Insulin	144 (18.0)
Metformin	618 (77.1)
Echocardiographic parameters	
GLS (%)	−18.0 [-19.3, −16.7]
E/e’	9.5 [7.8, 12]
E/A	0.8 [0.68, 0.99]
e’(cm/s)	7.0 [5.2, 8]
LAVi (ml/m^2^)	32.7 [27.7, 39]
LVMi (g/m^2^)	75.7 [64.1, 88.5]
Risk scores	
ARIC-HF 4 year (%)	8 [4, 12]
WATCH-DM (points)	11 [9, 13]

*ACEi* angiotensin converting enzyme inhibitor, *ARB* angiotensin receptor blocker, *BMI* body mass index, *CCB* calcium channel blocker, *CKD* chronic kidney disease, *DBP* diastolic blood pressure, *eGFR* estimated glomerular filtration rate, *GLS* global longitudinal strain, *HbA1c* haemoglobin A1 concentration, *HDL* high-density lipoprotein, *hs-TnT* high sensitivity troponin-T, *LAVi* left atrial volume indexed to body surface area, *LVMi* left ventricular mass indexed to body surface area, *MI* myocardial infarction, *SBP* systolic blood pressure, *T2DM* type 2 diabetes mellitus, *WHR* waist-to-hip ratio

### Echocardiographic parameters and phenotypic subtypes

Overall, systolic echocardiographic parameters for the cohort were within the normal range (Table 1). Diastolic parameters indicated mildly impaired LV relaxation (median e’ 7.0 [IQR 5.2–8]). The numbers of participants with abnormal systolic, diastolic and LV geometry parameters are shown in Table 2. There were higher numbers with abnormal LV relaxation (e’ > 8 cm/s, n = 629 [78%]) than participants with a dilated left atrium (LAVi > 34 ml/m^2^, n = 113 [44%]).

**Table 2:** Number of participants with an abnormal echocardiographic parameter (n = 804)

Echocardiographic parameters	n (%)
GLS ≥ -16%	126 (17%)
E/e’ ≥ 14	114 (15%)
e’ < 8 cm/s	629 (78%)
LAVi > 34 ml/m^2^	351 (44%)
LVMi > 88 g/m^2^ (female); 102 g/m^2^ (male)	113 (14%)
Clinical biomarker	
NTpBNP > 125 pg/mL	151 (19%)
hs-TnT > 12.5 pg/mL	217 (31%)

*GLS* global longitudinal strain, *LAVi* left atrial volume indexed to body surface area, *LVMi* left ventricular mass indexed to body surface area

**Fig. 1:**
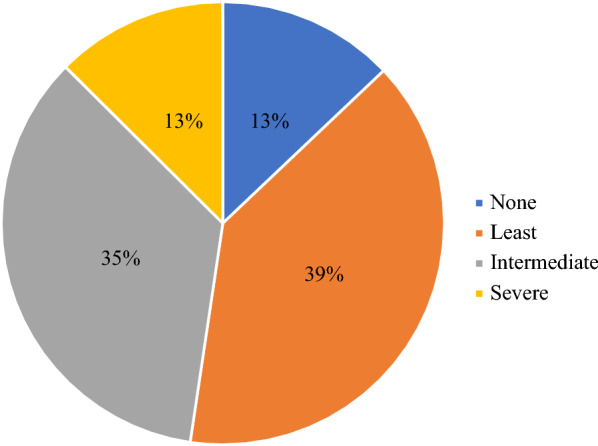
Diabetic cardiomyopathy subtypes at baseline (n = 804). None = no abnormal echocardiographic parameter. Least restrictive = 1 abnormal echocardiographic parameter (diastolic impairment, dilated left atrium or increase LV mass). Intermediate restrictive = at least 2 abnormal echocardiographic parameters and NTpBNP level within the normal range (< 125 pg/mL). Severe = at least 2 abnormal echocardiographic parameters and an elevated NTpBNP level (≥ 125 pg/mL)

DCM phenotypes, based on diastolic function and LV geometry on echocardiography, as well as NTpBNP levels, are explored in Fig. 1. The most observed phenotypes conformed to the “least restrictive” phenotype (39% of participants) and intermediate restrictive phenotypes (35%). Only 15% were observed to have a severely restrictive phenotype due to the presence of at least 2 abnormal echocardiographic parameters and an elevated NTpBNP level (defined as ≥ 125 pg/mL).

Increasing thresholds of NTpBNP were associated with longer diabetes duration. Participants with NTpBNP above 125 pg/mL had longer DM duration than those below this level (12 [IQR 6.5–19] vs. 10 [5–16], *p* = 0.027), and this became more prominent at higher cutoffs of 150 pg/mL (13 IQR [8–19] vs. 10 [5–16], *p* = 0.003). Likewise, the most severe DCM phenotype had longer DM duration than those with the mild and intermediate phenotypes (14 [IQR 8–20] vs. 10 [5–16], *p* = 0.007). However, an analysis of the association of abnormal findings over increasing duration of diabetes (Tables 3 and 4) showed only the proportion of patients with abnormal E/e’ to increase with diabetes duration.

**Table 3:** Association of diabetes duration with various cut-points on biomarker and echocardiographic tests

Parameter	Cut point	DM duration below cut point (years)	DM duration above cut point (years)	p
NTpBNP (pg/mL)	< 100	10 [5, 16]	10 [6, 17]	0.189
	100–124.9	10 [5, 16]	11.5 [6, 18]	0.098
	125–149.9	10 [5, 16]	12 [6.5, 19]	0.027
	150–174.9	10 [5, 16]	13 [8, 19]	0.003
	> 175	10 [5, 16]	13 [8, 19]	0.007
DCM phenotype	Severe	10 [5, 16]	14 [8, 19]	0.007

*DCM* diabetic cardiomyopathy; DM = diabetes mellitus

**Table 4:** Prevalence of abnormal imaging and biomarker results according to diabetes duration

Quartile; Diabetes duration (y)	n	NTpBNP 125 pg/mL n (%)	GLS ≥ -16% n (%)	E/e’ ≥ 14 n (%)	e’ < 8 cm/s n (%)	LAVi > 34 ml/m^2^ n (%)	LVMi > 88 g/m^2^ (F), > 102 g/m^2^ (M) n (%)
< 6	206	28 (13)	36 (18)	23 (11)	143 (67)	94 (44)	32 (15)
7–10	164	26 (15)	25 (16)	18 (11)	117 (68)	70 (41)	26 (15)
11–16	177	27 (15)	24 (14)	29 (16)	127 (70)	75 (41)	19 (10)
> 16	175	39 (21)	36 (20)	45 (23)	143 (73)	102 (52)	30 (15)
*p* value		0.194	0.494	0.001	0.543	0.095	0.473

*GLS* global longitudinal strain, *LAVi* left atrial volume indexed to body surface area, *LVMi* left ventricular mass indexed to body surface area

### Association of risk assessment tools with echocardiographic parameters

The relationship between the risk scores and clinical biomarkers with the echocardiographic parameters were explored in standardised linear regression analyses (Table 5). The ARIC-HF score was associated with a modest increase in GLS with each standard deviation increment in the score (standardised β = 0.22, *p* < 0.001) but only small changes in the other echocardiographic parameters. The WATCH-DM score had a similar increase with each parameter. In multivariable regression adjusted for age, BMI, and renal function (eGFR), NTpBNP showed modest increases in GLS, LV filling pressure (E/e’) and LAVi and similar trends were observed with hs-TnT. Associations of echocardiographic parameters according to gender were evaluated in the regression analysis and are shown in Additional file 3: Appendix S3. This showed greater association between GLS with the risk scores and biomarkers in males compared to females.

**Table 5:** The effect of 1 standard deviation increase in risk scores or clinical biomarkers on echocardiographic parameters

Variable	Standardized β, *p*-value				R^2^			
	ARIC-HF^	WATCH-DM^	NTpBNP*	hs-TnT*	ARIC-HF^	WATCH-DM^	NTpBNP*	hs-TnT*
GLS	0.22, *p* < 0.001	0.22, *p* < 0.001	0.32, *p* < 0.001	0.26, *p* < 0.001	0.05	0.05	0.18	0.14
E/e’	0.09, *p* = 0.015	0.33, *p* < 0.001	0.32, *p* < 0.001	0.29, *p* < 0.001	0.01	0.11	0.17	0.16
e’	−0.07, *p* = 0.060	−0.27, *p* < 0.001	−0.12, *p* = 0.001	−0.15, *p* = 0.001	0.005	0.08	0.08	0.09
LAVi	0.01, *p* = 0.813	0.23, *p* < 0.001	0.22, *p* < 0.001	0.15, *p* = 0.001	0.0001	0.04	0.10	0.06
LVMi	0.09, *p* = 0.014	0.23, *p* < 0.001	0.15, *p* < 0.001	0.24, *p* < 0.001	0.008	0.05	0.07	0.09

^univariable linear regression analysis, *multivariable linear regression analysis adjusted for age, body mass index and renal function

95% CI = 95% confidence interval; GLS = global longitudinal strain; hs-TnT = high sensitivity troponin-T; LAVi = left atrial volume indexed to body surface area; LVMi = left ventricular mass indexed to body surface area

### Sensitivity and specificity

**Fig. 2:**
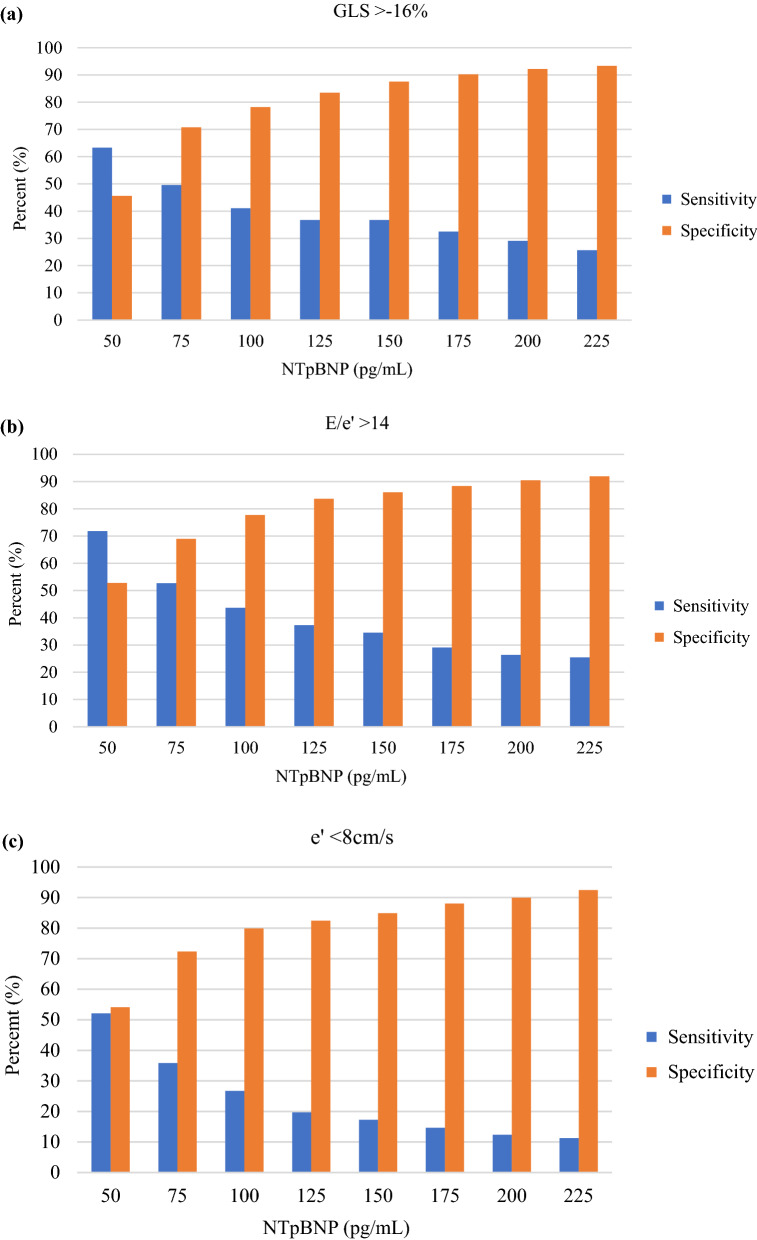

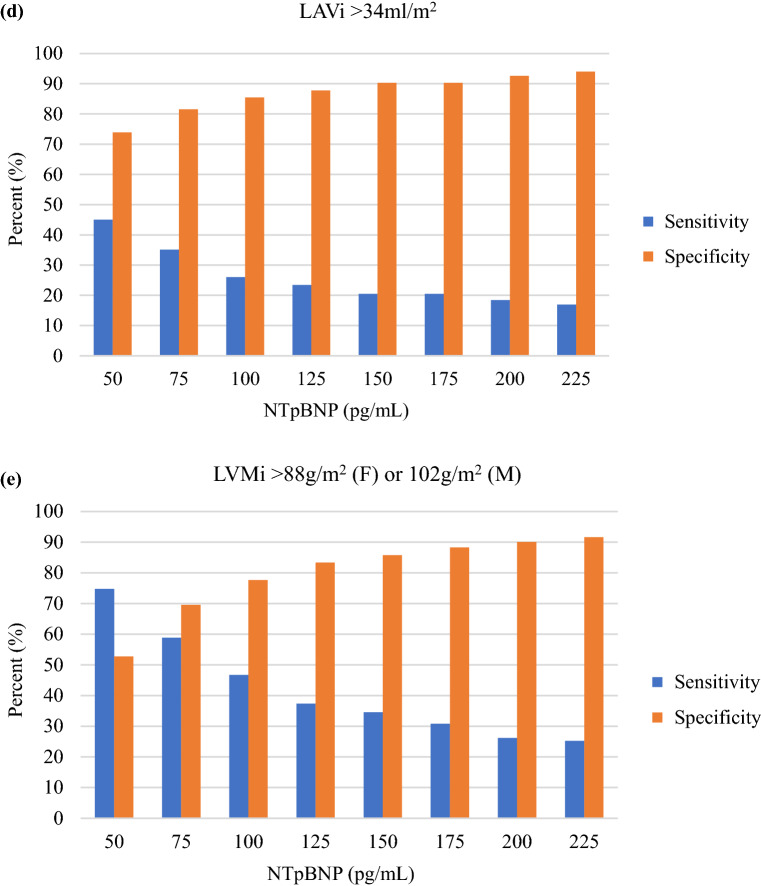
Sensitivity and specificity for the detection of abnormal echocardiographic parameters at different cut-points of NTpBNP (pg/mL). GLS = global longitudinal strain; LAVi = left atrial volume indexed to body surface area; LVMi = left ventricular mass indexed to body surface area; NTpBNP = N-terminal pro-brain natriuretic peptide

The sensitivity and specificity at different NTpBNP cut points are shown in Fig. 2. Even at the lowest cut-off (50 pg/ml), the sensitivity of NTpBNP for the detection of an abnormal GLS (Fig. 2a) was < 70%, with a low specificity. NTpBNP had modest ability to detect elevated LV filling pressures (E/e’ > 14; Fig. 2b). The discriminative ability to detect impaired LV relaxation was poor (e’ < 8 cm; Fig. 2c). Furthermore, the detection of increased LAV and LVM had a similar trend to detection of abnormal diastolic function (Fig. 2d and e).

A 90% cut-point for sensitivity to detect abnormal echocardiographic parameters was associated with a low-risk score and clinical biomarker level (Table 6). This corresponded with a low specificity indicating that the risk assessment tools have limited ability to detect abnormal echocardiographic parameters in T2DM. The ROC curves are shown in the Additional file 2: Appendix S2 and the AUC for each sub-population are shown in Additional file 4: Appendix S4.

**Table 6:** Risk assessment levels at 90% sensitivity with corresponding specificity

Test	Risk assessment tool	Echocardiographic parameter				
		GLS ≥ -16%	E/e’ > 14	e’ < 8 cm/s	LAVi > 34 ml/m^2^	LVMi > 88 g/m^2^ (F) or 102 g/m^2^ (M)
Cut point for 90% sensitivity	ARIC-HF (%)	3	5	3	3	4
	WATCH-DM (points)	8	10	8	8	9
	NTpBNP (pg/mL)	13	29	13	20	18
	hs-TnT (pg/mL)	6	6	5	6	6
AUC	ARIC-HF	63%	59%	54%	52%	59%
	WATCH-DM	63%	74%	60%	59%	65%
	NTpBNP	63%	69%	55%	65%	67%
	hs-TnT	63%	68%	61%	59%	64%
	*p*-value	0.948	< 0.001	0.452	< 0.001	0.599
Specificity at the 90% cut point for sensitivity	ARIC-HF	11%	29%	13%	12%	19%
	WATCH-DM	12%	38%	15%	12%	22%
	NTpBNP	10%	32%	13%	23%	16%
	hs-TnT	20%	19%	18%	22%	20%
	*p*-value	0.075	0.173	0.810	0.104	0.749

*GLS* global longitudinal strain, *hs-TnT* high sensitivity troponin-T, *LAVi* left atrial volume indexed to body surface area, *LVMi* left ventricular mass indexed to body surface area, *NTpBNP* N-terminal pro-brain natriuretic peptide

## Discussion

In this analysis of community-based studies of people with T2DM, many of whom had HF risk factors, the prevalence of subclinical LVD and SHD varied according to the parameter selected from 14–78%. The most common DCM phenotypes were the least and intermediate restrictive patterns without elevated biomarker levels. A longer duration of diabetes was associated with the severe DCM phenotype. Results of our study indicate that NTpBNP and hs-TnT had minimal correlation to underlying LVD in asymptomatic T2DM. As a screening strategy these clinical biomarkers had poor discriminative ability to detect subclinical LVD and SHD. Furthermore, the sensitivity and specificity of both biomarkers and clinical scores were modest for the detection of abnormal systolic, diastolic and LV geometric parameters.

### Subclinical LVD detection strategies in T2DM

HF is an established and well-known complication of diabetes; however, its true prevalence is probably under-appreciated [12]. Several key factors influence its development, including female sex [13] and poor glycaemic control [14]. HF is a chronic and progressive disease, and symptoms emerge late in its course. Detection of the early stages might allow interventions to delay or prevent progression. The ACC/AHA/HFSA HF guidelines describe the progression though HF stages [1]. Delineating HF in this way underlines the importance of early detection to reduce its progression to symptomatic HF (stage C and D).

Risk assessment scores to estimate the probability of HF have been developed in various populations. Risk scores are a desirable means to evaluate HF risk as they are largely based on clinical parameters, are easy to use and come at no cost. The ARIC-HF risk score was developed in community-based patients with reasonable prediction of HF (AUC = 0.7937) [3]. In populations of T2DM, the ARIC-HF score was elevated in people that developed clinical HF compared to those that did not [15]. The WATCH-DM score was specifically developed in asymptomatic people with T2DM [4], and incorporates several clinical enhancing factors that increase the risk of HF in T2DM. It has been validated in several independent cohorts [16], involving patients at a similar risk level to our populations, among whom incident HF developed in 2–4% over 5 years follow-up.

Recently published guidelines from the American Diabetes Association have highlighted detection strategies used for asymptomatic (stage B) HF [10]. They recommend the use of clinical biomarkers (BNP, NTpBNP and high-sensitivity troponin) to determine the need for further imaging, such as echocardiography. This recommendation was largely based on previously published reports of the use of clinical biomarkers to predict clinical HF [6–9]. The STOP-HF study used a BNP-guided strategy to further investigate and manage subclinical LVD in asymptomatic people aged over 40 years with at least 1 risk factor for HF (only 20% had T2DM) [5]. In the overall cohort, the primary endpoint of LVD or HF events was reduced in the BNP-guided group compared to usual care (unadjusted OR 0.55 [95% CI 0.37, 0.82], *p* = 0.001). As expected, there was a greater uptake of pharmacotherapy such RAAS-modifying therapies in the BNP-guided group. Although not specifically targeted to T2DM, this study demonstrates that effective treatment strategies in early HF can reduce its progression.

### Association of biomarkers with diabetic cardiomyopathy phenotypes

The pathophysiology of DCM is complex due to phenotypic variation and the interplay of several dysregulated pathways that lead to the cardiomyopathy. Both ischemic and non-ischemic etiologies are important, and although the latter reflect myocardial disease, the existence of different phenotypic clusters [17] suggest that a number of mechanisms are contributing. It seems improbable that a single biomarker strategy will capture these equally.

Isolated echocardiographic variants of LV structure and function are commonly seen in asymptomatic T2DM [15]. However, detection of LVD is reliant on parameters assessed. In a study evaluating exercise intolerance in T2DM by cardiopulmonary exercise testing, those with effort intolerance had reduced peripheral oxygen consumption and impaired GLS despite normal LVEF highlighting the higher sensitivity of GLS to detect subclinical LVD [18]. In our study, despite the presence of normal ejection fraction, abnormal GLS was documented in 17% of patients in this study. Impaired GLS indicates subclinical systolic impairment [19], and is prognostically important [15]. However, this signal was only weakly associated with both clinical scores and biomarkers.

Diastolic dysfunction (DD) can be appreciated in the early stages of the disease [20]. Although nearly 80% of asymptomatic patients with T2DM showed impaired relaxation in this study, LA enlargement was present in 44% and only 15% showed increased E/e’. Thus, most DD in these asymptomatic patients was mild, although a minority showed progression to elevated LV filling pressures and associated adverse outcomes [21, 22]. Diastolic markers were better associated with the WATCH-DM than the ARIC score, and evidence of raised filling pressure was associated with NTpBNP or Tn. The third phenotypic manifestation of DCM involves changes in LV geometry, reflecting remodelling and fibrosis [23]. Increased LVMi was the most uncommon abnormality, and was weakly associated with the clinical scores and biomarkers.

These results provide further insight into the LV functional and geometric changes seen in early HF, and emphasize that phenotypic variations in DCM are observed in asymptomatic patients. Elevated biomarkers in asymptomatic patients [11] are most strongly associated with abnormal GLS and E/e’, albeit accounting for < 20% of the variance of these parameters. In contrast, markers of isolated myocardial disease (e’) are less strongly associated with biomarkers. Combinations of markers of LV dysfunction may highlight the progressive nature of the disease. Nonetheless, the development of subclinical LVD is not a benign state, as previous studies have shown that even one abnormal echocardiographic parameter is associated with reduced survival—suggesting the need for detection and early intervention [15]. Reliance on biomarkers to select patients for echocardiography may limit the recognition of DCM.

### Implications for screening for subclinical LVD in T2DM

Screening for stage B HF is vitally important in patients with T2DM as the burden of the disease is likely to increase. The results of this study add the components of timing and disease severity to screening for LV dysfunction in T2DM. Furthermore, this study demonstrates that when used in isolation, currently available clinical risk scores and biomarkers cannot reliably predict prevalent stage B HF in T2DM.

Clearly, comprehensive echocardiography is neither a feasible nor a cost-effective means of responding to HF risk at the population level. Future screening algorithms may enhance detection by incorporating various facets of clinical parameters and biomarkers to improve the sensitivity of detection of underlying LVD or SHD. However, handheld cardiac ultrasound (HHU) may offer an alternative strategy for screening. In a recently published meta-analysis, detection rates of abnormal LV indices, such as LV dilatation, wall motion abnormalities, reduced ejection fraction and hypertrophy with HHU were high [24]. But as expected, detection rates were superior in experienced users compared to inexperienced users, such as primary care physicians and nurses [24]. Given the burden of T2DM remains within the community, improved diagnostic accuracy with the inexperienced user is therefore desirable. Coupling artificial intelligence with HHU may resolve this issue. In fact, detection of abnormal LV function by an automated ejection fraction algorithm in HHU was high (90% sensitivity and 87% specificity) in a real-world patient population [25] suggesting its feasibility for use in widespread screening. Although attractive alternatives, future research in this area is needed to determine the usefulness of HHU in high-risk HF groups, such as T2DM.

### Study limitations

Recruitment into the 3 studies was performed by advertisement and invitation letters which may inherently lead to selection and volunteer bias. Numerous risk scores have been reported for the prediction of HF, and only two were tested here, albeit the most widely used and specific to DM. The discrimination of the various tools (as measured by the AUCs) vary between the cohorts, but it seems unlikely that the overall conclusion about the inconsistency of biomarker and physiologic data would be the different if any single cohort were considered alone. Finally, prognostic data was not included in this analysis due to limited data within our cohort.

## Conclusion

Although biomarkers have a greater accessibility and lower cost than echocardiography, their use may not permit appropriate testing and management of many patients with subclinical LVD or SHD in T2DM.

## Supplementary Information

**Additional file 1: Table S1. **Baseline demographic data for each population studied.

**Additional file 2: Figure S1. **Discriminative ability for NTpBNP to detect abnormal echocardiographic parameters. **Figure S2. **Discriminative ability for hs-TnT to detect abnormal echocardiographic parameters. **Figure S3. **Discriminative ability for the ARIC-HF score to detect abnormal echocardiographic parameters. **Figure S4. **Discriminative ability for the WATCH-DM score to detect abnormal echocardiographic parameters.

**Additional file 3: Table S3. **The effect of 1 standard deviation increase in risk scores or clinical biomarkers on echocardiographic parameters by gender.

**Additional file 4: Table S4. **Area under the receiver operator curve for each risk assessment tool to detect an abnormal echocardiographic parameter in each sub-population.

## Data Availability

The investigators and are open to requests for collaboration. Interested parties can approach the corresponding author regarding availability of data and materials.

## Abbreviations

ARIC: Atherosclerotic Risk In Communities
DCM: Diabetic cardiomyopathy
GLS: Global longitudinal strain
HbA1c: Haemoglobin A1c
HF: Heart failure
hs-TnT: High sensitivity troponin-T
LAV: Left atrial volume
LVD: Left ventricular dysfunction
LVM: Left ventricular mass
NTpBNP: N-terminal pro-brain natriuretic peptide
T2DM: Type 2 diabetes mellitus
