# Supplementary material for: Association of biomarkers and risk scores with subclinical left ventricular dysfunction in patients with type 2 diabetes mellitus
Source: Cardiovasc Diabetol. 2022 Dec 9;21:278. doi: 10.1186/s12933-022-01711-5 (PMC9737699; doi:10.1186/s12933-022-01711-5)
Supplement: Supplementary file 1 — Additional file 1: Table S1. Baseline demographic data for each population studied. [file 12933_2022_1711_MOESM1_ESM.docx]

**Additional file Materials**

**Additional file 1 Table S1.** Baseline demographic data for each population studied.

| **Variable** | **Median [IQR] or n (%)** | | |
| --- | --- | --- | --- |
|  | VicELF  n = 112 | TasELF  n = 135 | PREDICT  n = 557 |
| Age (years) | 70 [68-73] | 71 [67-74] | 68 [62-72] |
| Female gender | 51 (46) | 62 (46) | 174 (31) |
| Overweight and obese | 103 (93) | 118 (87) | 473 (85) |
| Prior MI | 0 | 5 (4) | 57 (10) |
| Prior stroke | 9 (8) | 11 (8) | 28 (5) |
| History of smoking (current & ex) | 52 (46) | 71 (54) | 263 (47) |
| BMI | 29.7 [26.9-33.3] | 28.6 [26.2-31.8] | 29.4 [26.3-33.4] |
| WHR | 0.96 [0.91-1.01] | 0.97 [0.91-1.02] | 0.98 [0.93-1.03] |
| SBP (mmHg) | 141 [130-153] | 138 [128-145] | 127 [117-138] |
| DBP (mmHg) | 85 [78-90] | 81 [76-87] | 71 [65-78] |
| HbA1c (%) | 6.5 [5.8-7.4] | 6.8 [6.3-7.7] | 7.1 [6.5-7.8] |
| Creatinine (mmol/L) | 74 [66-86] | 74.5 [65-99] | 76 [64-91] |
| eGFR (ml/min/1.73m^2^) | 81 [70-88] | 79 [63-89] | 86 [72-95] |
| HDL (mg/dL) | 49 [41-57] | 49 [41-57] | 46 [39-54] |
| NTpBNP (pg/mL) | 44 [28-76] | 66 [29-157] | 49 [24-101] |
| hs-TnT | 6.2 [5.3-7.6] | 8.8 [6.4-13.4] | 9.6 [6.9-13.9] |
| **Medications** | |  |  |
| Beta-blocker | 17 (15) | 3 (4) | 97 (17) |
| ACEi/ARB | 80 (71) | 101 (75) | 361 (65) |
| Diuretics | 33 (29) | 11 (8) | 115 (21) |
| CCB | 42 (38) | 33 (24) | 147 (26) |
| Statin | 87 (78) | 68 (50) | 397 (71) |
| Anti-platelet | 31 (28) | 45 (33) | 219 (39) |
| Insulin | 8 (7) | 33 (25) | 103 (18) |
| Metformin | 84 (75) | 87 (65) | 447 (80) |
| **Echocardiographic parameters** | |  |  |
| GLS (%) | -18.3 [-20- -16.7] | -17.6 [-19- -16.2] | -18.3 [-19- -16.7] |
| E/e’ | 8.8 [7.5-10.8] | 8.7 [7.3-10.4] | 9.8 [8.2-12.6] |
| E/A | 0.76 [0.68-0.93] | 0.8 [0.67-0.72] | 0.8 [0.69-1.05] |
| e’(cm/s) | 7.4 [6.3-8.6] | 7.9 [6.6-8.7] | 6 [5-8] |
| LAVi (ml/m^2^) | 33.0 [28-40] | 28.3 [24-34] | 33.6 [28.5-40] |
| LVMi (g/m^2^) | 70.1 [57-83] | 79.2 [67-95] | 76 [64-88] |
| **Risk score** | | | |
| ARIC-HF 4 year (%) | 10 [7-15] | 8 [5-12] | 7 [4-12] |
| WATCH-DM (points) | 10 [9-11] | 10 [9-12] | 11 [9-13] |

ACEi = angiotensin converting enzyme inhibitor; ARB = angiotensin receptor blocker; BMI = body mass index; CCB = calcium channel blocker; CKD = chronic kidney disease; DBP = diastolic blood pressure; eGFR = estimated glomerular filtration rate; GLS = global longitudinal strain; HbA1c = haemoglobin A1 concentration; HDL = high-density lipoprotein; hs-TnT = high-sensitivity troponin-T; LAVi = left atrial volume indexed to body surface area; LVMi = left ventricular mass indexed to body surface area; MI = myocardial infarction; SBP = systolic blood pressure; T2DM = type 2 diabetes mellitus; WHR = waist-to-hip ratio.
