# Supplementary material for: Association of biomarkers and risk scores with subclinical left ventricular dysfunction in patients with type 2 diabetes mellitus
Source: Cardiovasc Diabetol. 2022 Dec 9;21:278. doi: 10.1186/s12933-022-01711-5 (PMC9737699; doi:10.1186/s12933-022-01711-5)
Supplement: Supplementary file 2 — Additional file 2: Figure S1. Discriminative ability for NTpBNP to detect abnormal echocardiographic parameters. Figure S2. Discriminative ability for hs-TnT to detect abnormal echocardiographic parameters. Figure S3. Discriminative ability for the ARIC-HF score to detect abnormal echocardiographic parameters. Figure S4. Discriminative ability for the WATCH-DM score to detect abnormal echocardiographic parameters. [file 12933_2022_1711_MOESM2_ESM.docx]

**Additional file Materials**

**Additional file 2: Figure S1.** Discriminative ability for NTpBNP to detect abnormal echocardiographic parameters.

| 1. GLS ≥-16%   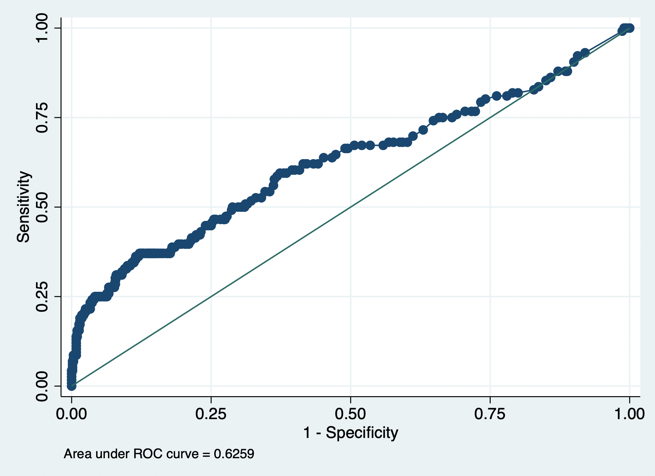 | 1. E/e’ >14   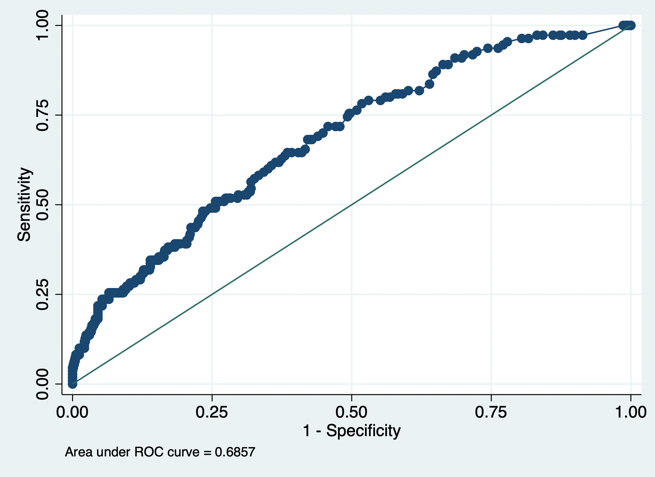 |
| --- | --- |
| 1. e’ <8cm/s   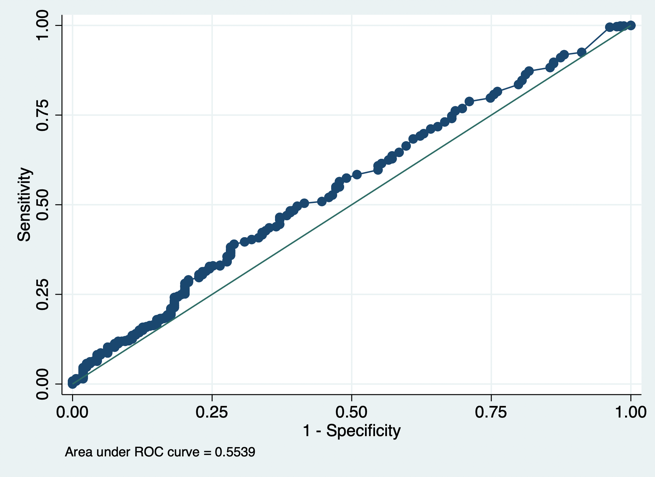 | 1. LAVi >34ml/m^2^   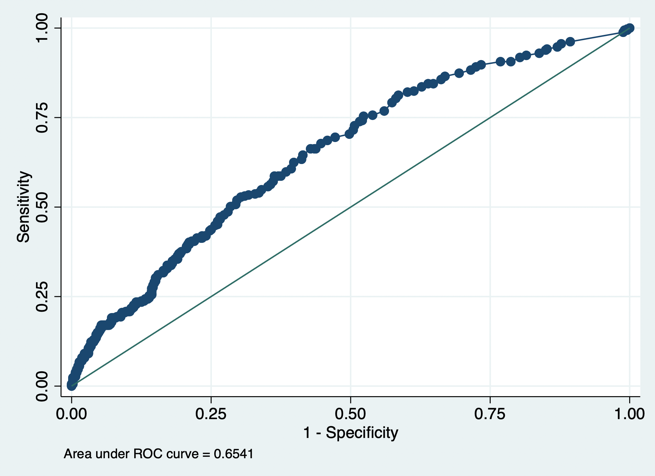 |
| 1. LVMi >88g/m^2^ (F) or 102g/m^2^ (M)   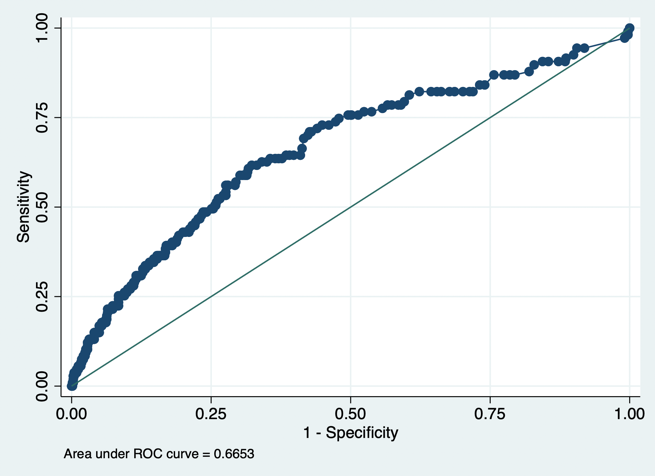 |  |

GLS = global longitudinal strain; LAVi = left atrial volume indexed to body surface area; LVMi = left ventricular mass indexed to body surface area; NTpBNP = N-terminal pro-brain natriuretic peptide.

**Additional file 2: Figure S2.** Discriminative ability for hs-TnT to detect abnormal echocardiographic parameters.

| 1. GLS ≥-16%   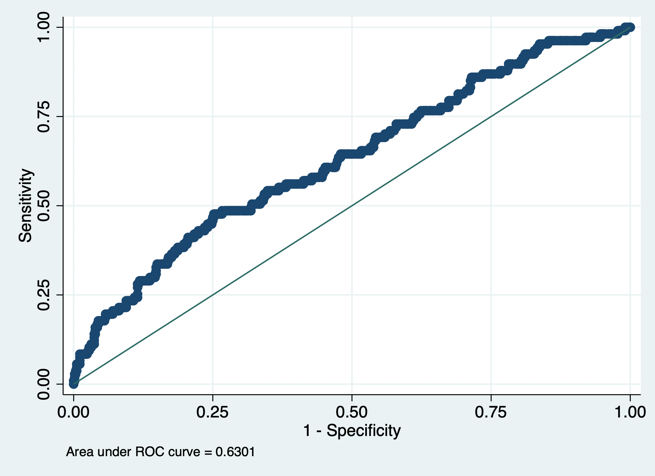 | 1. E/e’ >14   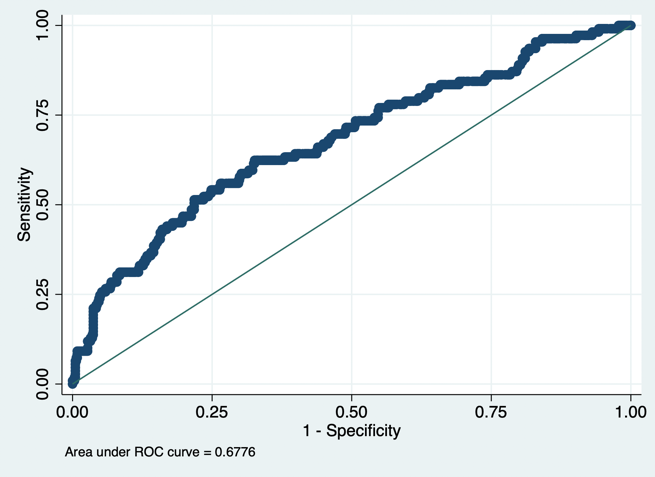 |
| --- | --- |
| 1. e’ <8cm/s   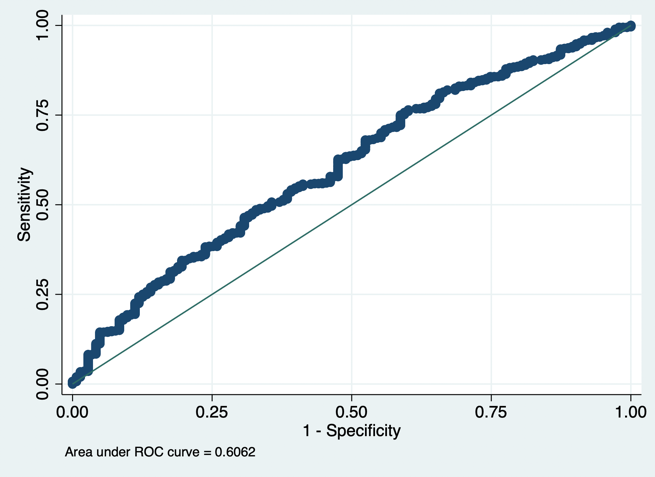 | 1. LAVi >34ml/m^2^   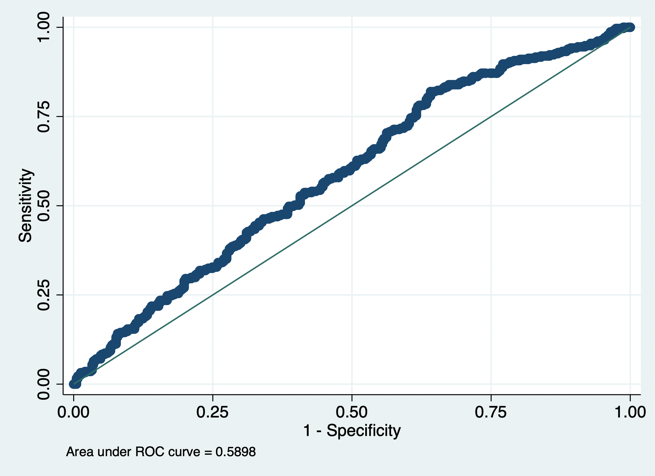 |
| 1. LVMi >88g/m^2^ (F) or 102g/m^2^ (M)   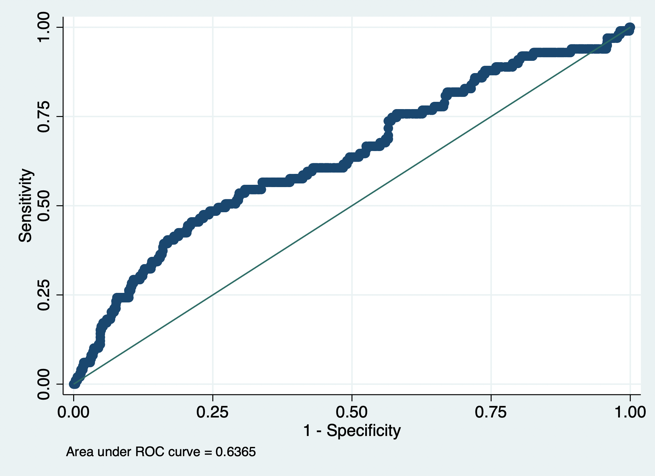 |  |

GLS = global longitudinal strain; LAVi = left atrial volume indexed to body surface area; LVMi = left ventricular mass indexed to body surface area; hs-TnT = high sensitivity troponin-T.

**Additional file 2: Figure S3.** Discriminative ability for the ARIC-HF score to detect abnormal echocardiographic parameters.

| 1. GLS ≥-16%   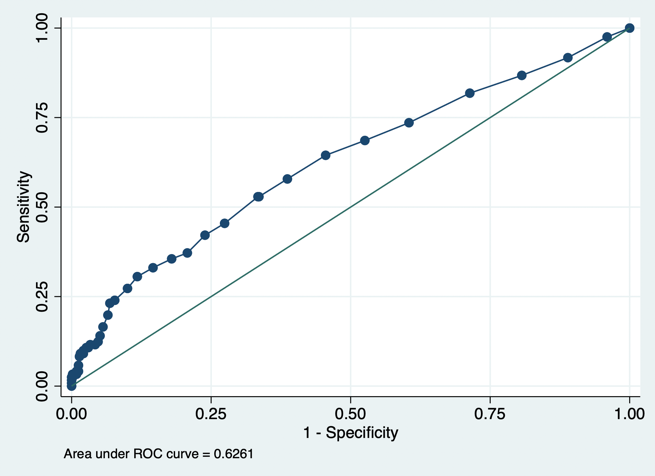 | 1. E/e’ >14   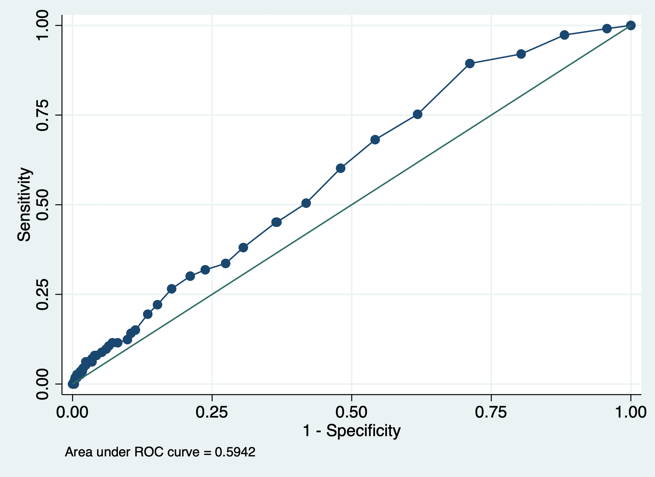 |
| --- | --- |
| 1. e’ <8cm/s   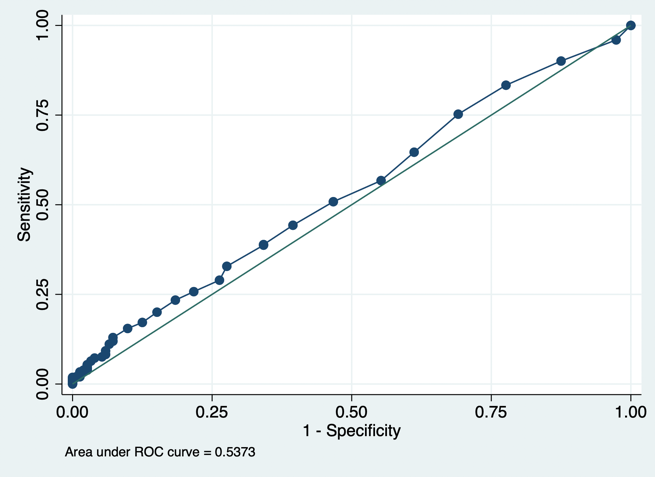 | 1. LAVi >34ml/m^2^   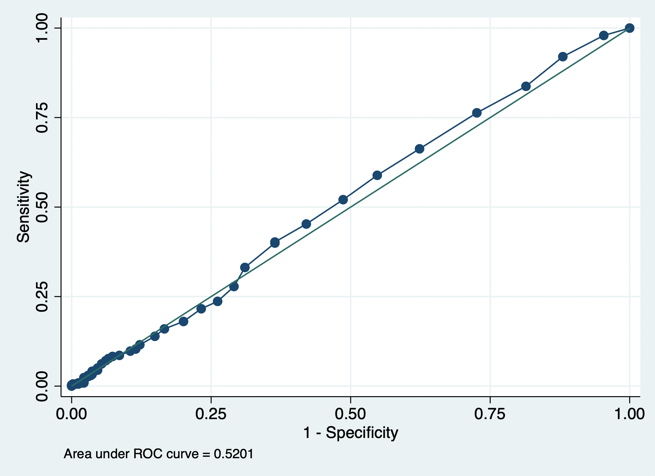 |
| 1. LVMi >88g/m^2^ (F) or 102g/m^2^ (M)   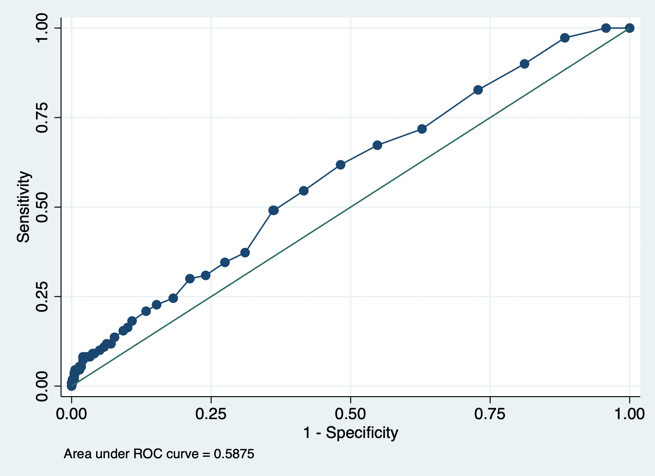 |  |

GLS = global longitudinal strain; LAVi = left atrial volume indexed to body surface area; LVMi = left ventricular mass indexed to body surface area.

**Additional file 2: Figure S4.** Discriminative ability for the WATCH-DM score to detect abnormal echocardiographic parameters.

| 1. GLS ≥-16%   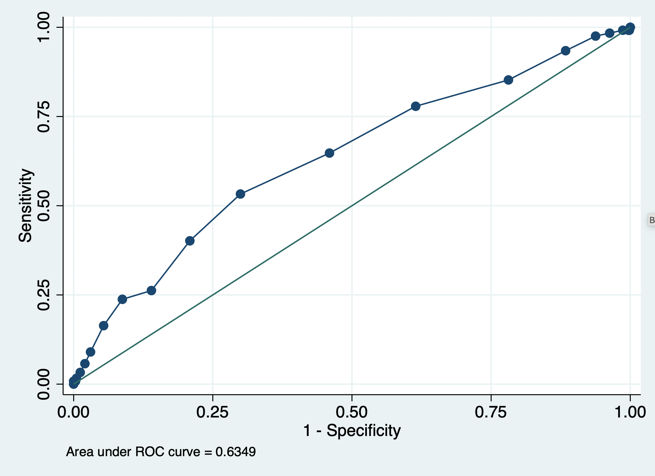 | 1. E/e’ >14   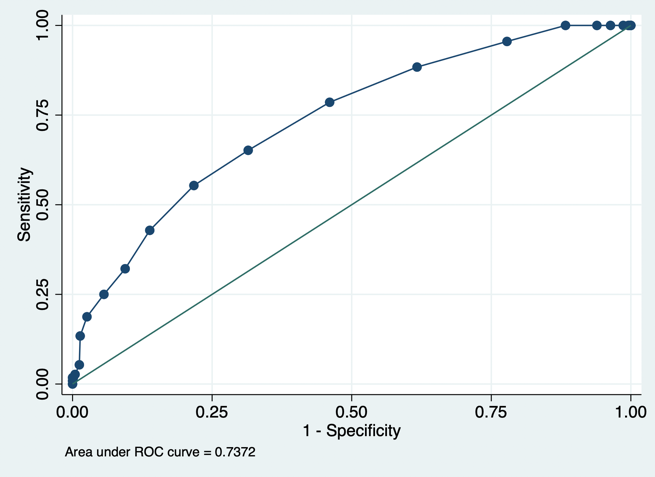 |
| --- | --- |
| 1. e’ <8cm/s   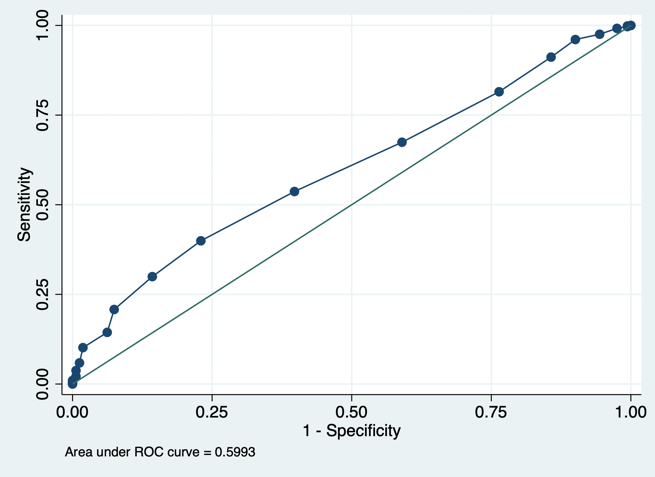 | 1. LAVi >34ml/m^2^   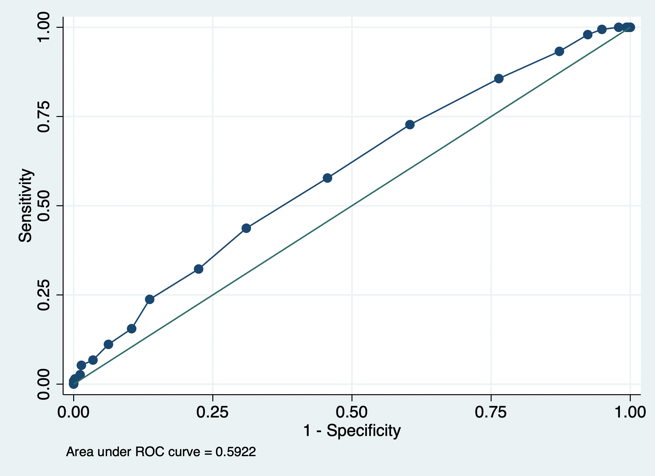 |
| 1. LVMi >88g/m^2^ (F) or 102g/m^2^ (M)   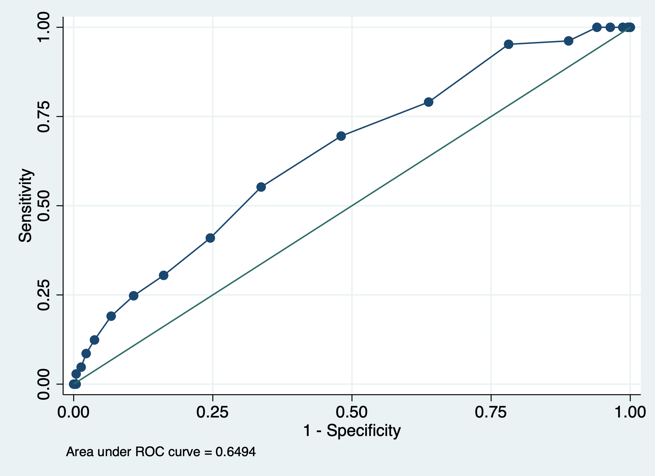 |  |

GLS = global longitudinal strain; LAVi = left atrial volume indexed to body surface area; LVMi = left ventricular mass indexed to body surface area.
