# Supplementary material for: Association of biomarkers and risk scores with subclinical left ventricular dysfunction in patients with type 2 diabetes mellitus
Source: Cardiovasc Diabetol. 2022 Dec 9;21:278. doi: 10.1186/s12933-022-01711-5 (PMC9737699; doi:10.1186/s12933-022-01711-5)
Supplement: Supplementary file 3 — Additional file 3: Table S3. The effect of 1 standard deviation increase in risk scores or clinical biomarkers on echocardiographic parameters by gender. [file 12933_2022_1711_MOESM3_ESM.docx]

**Additional file Materials**

**Additional file 3: Table S3.** The effect of 1 standard deviation increase in risk scores or clinical biomarkers on echocardiographic parameters by gender.

| **Variable** | **Standardized β, *p*-value** | | | | **R^2^** | | | |
| --- | --- | --- | --- | --- | --- | --- | --- | --- |
|  | **ARIC-HF^** | **WATCH-DM^** | **NTpBNP*** | **hs-TnT*** | **ARIC-HF^** | **WATCH-DM^** | **NTpBNP*** | **hs-TnT*** |
| Female (n=287) | | | | | | | | |
| GLS | 0.09, *p=*0.163 | 0.12, *p=*0.065 | 0.11, *p*=0.144 | 0.35, *p*<0.001 | 0.01 | 0.05 | 0.03 | 0.14 |
| E/e’ | 0.10, *p*=0.090 | 0.33, *p*<0.001 | 0.20, *p=*0.005 | 0.25, *p=*0.001 | 0.01 | 0.11 | 0.12 | 0.15 |
| e’ | -0.01, *p*=0.825 | -0.23, *p*<0.001 | 0.05, *p*=0.444 | -0.01, *p*=0.901 | 0.002 | 0.05 | 0.08 | 0.10 |
| LAVi | 0.02, *p*=0.735 | 0.18, *p=*0.002 | 0.28, *p*<0.001 | 0.05, *p*=0.518 | 0.0004 | 0.03 | 0.14 | 0.08 |
| LVMi | 0.05, *p*=0.395 | 0.19, *p=*0.001 | 0.08, *p=*0.233 | 0.03, *p=*0.673 | 0.003 | 0.04 | 0.06 | 0.08 |
| Male (n=517) | | | | | | | | |
| GLS | 0.29, *p*<0.001 | 0.27, *p*<0.001 | 0.24, *p*<0.001 | 0.20, *p=*0.001 | 0.07 | 0.05 | 0.10 | 0.09 |
| E/e’ | 0.10, *p*=0.033 | 0.37, *p*<0.001 | 0.19, *p*<0.001 | 0.35, *p*<0.001 | 0.01 | 0.14 | 0.10 | 0.17 |
| e’ | -0.09, *p*=0.044 | -0.30, *p*<0.001 | -0.07, *p*=0.140 | -0.21, *p<*0.001 | 0.01 | 0.09 | 0.08 | 0.09 |
| LAVi | -0.01, *p*=0.773 | 0.20, *p*<0.001 | 0.20, *p*<0.001 | 0.17, *p*=0.003 | 0.0002 | 0.04 | 0.07 | 0.05 |
| LVMi | 0.08, *p*=0.091 | 0.22, *p*<0.001 | 0.19, *p*<0.001 | 0.26, *p*<0.001 | 0.006 | 0.05 | 0.06 | 0.08 |

^ = univariable linear regression analysis. * = multivariable linear regression analysis adjusted for age, body mass index and renal function.

95% CI = 95% confidence interval; GLS = global longitudinal strain; hs-TnT = high sensitivity troponin-T; LAVi = left atrial volume Indexed to body surface area; LVMi = left ventricular mass indexed to body surface area.
