# Supplementary material for: Association of biomarkers and risk scores with subclinical left ventricular dysfunction in patients with type 2 diabetes mellitus
Source: Cardiovasc Diabetol. 2022 Dec 9;21:278. doi: 10.1186/s12933-022-01711-5 (PMC9737699; doi:10.1186/s12933-022-01711-5)
Supplement: Supplementary file 4 — Additional file 4: Table S4. Area under the receiver operator curve for each risk assessment tool to detect an abnormal echocardiographic parameter in each sub-population. [file 12933_2022_1711_MOESM4_ESM.docx]

**Additional file Materials**

**Additional file 4: Table S4.** Area under the receiver operator curve for each risk assessment tool to detect an abnormal echocardiographic parameter in each sub-population.

| **Sub-population** | **Risk assessment tool** | **AUC** | | | | |
| --- | --- | --- | --- | --- | --- | --- |
|  |  | **GLS**  **≥-16%** | **E/e’**  **>14** | **e’**  **<8cm/s** | **LAVi**  **>34ml/m^2^** | **LVMi**  **>88g/m^2^ (F) or 102g/m^2^ (M)** |
| VicELF  n=112 | ARIC-HF | 62% | 48% | 60% | 57% | 58% |
|  | WATCH-DM | 49% | 58% | 56% | 64% | 62% |
|  | NTpBNP | 50% | 58% | 49% | 71% | 72% |
|  | hs-TnT | 71% | * | 66% | 63% | * |
|  | *p*-value | 0.030 | 0.381 | 0.004 | 0.685 | 0.416 |
| TasELF  n=135 | ARIC-HF | 63% | 66% | 57% | 48% | 53% |
|  | WATCH-DM | 59% | 67% | 51% | 60% | 57% |
|  | NTpBNP | 71% | 69% | 48% | 58% | 53% |
|  | hs-TnT | 60% | 59% | 53% | 52% | 58% |
|  | *p*-value | 0.676 | 0.221 | 0.398 | 0.279 | 0.903 |
| PREDICT  n=557 | ARIC-HF | 62% | 62% | 59% | 53% | 61% |
|  | WATCH-DM | 69% | 74% | 63% | 60% | 69% |
|  | NTpBNP | 63% | 70% | 62% | 69% | 71% |
|  | hs-TnT | 66% | 68% | 62% | 61% | 72% |
|  | *p*-value | 0.275 | 0.006 | 0.488 | <0.001 | 0.127 |

*=outcome does not vary.

GLS = global longitudinal strain; hs-TnT = high sensitivity troponin-T; LAVi = left atrial volume indexed to body surface area; LVMi = left ventricular mass indexed to body surface area; NTpBNP = N-terminal pro-brain natriuretic peptide.
